# Synthesis, Thermal Behavior and Mechanical Property of Fully Biobased Poly(hexamethylene Furandicarboxylate-*co*-hexamethylene Thiophenedicarboxylate) Copolyesters

**DOI:** 10.3390/polym17141997

**Published:** 2025-07-21

**Authors:** Haidong Yang, Shiwei Feng, Zhaobin Qiu

**Affiliations:** State Key Laboratory of Chemical Resource Engineering, Beijing University of Chemical Technology, Beijing 100029, China; 2023200346@grad.buct.edu.cn

**Keywords:** biobased copolyesters, poly(hexamethylene furandicarboxylate), poly(hexamethylene thiophenedicarboxylate), thermal behavior, structure–property relationship

## Abstract

In order to increase the toughness of poly(hexamethylene furandicarboxylate) (PHF) without severely compromising its strength at break, novel biobased poly(hexamethylene furandicarboxylate-*co*-hexamethylene thiophenedicarboxylate) (PHFTh) copolyesters and their parent homopolyesters, PHF and poly(hexamethylene thiophenedicarboxylate), were successfully synthesized through melt polycondensation in this research. Despite the variation in their compositions, all the PHFTh copolyesters exhibited excellent thermal stability. The PHFTh copolyesters were semicrystalline in nature, showing the lowest eutectic melting points and isodimorphism behaviors over the whole composition range. As the hexamethylene thiophenedicarboxylate (HTh) unit content increased, the glass transition temperature of the copolyesters gradually decreased, while the chain mobility was accordingly enhanced. Therefore, the introduction of the HTh unit significantly increased the elongation at break of the PHFTh, achieving a balance between strength and toughness. The biobased PHFTh copolyesters showed tunable thermal behaviors and excellent mechanical properties and may find potential end uses from a practical application viewpoint.

## 1. Introduction

The widespread utilization of disposable plastics has significantly enhanced daily convenience, yet concurrently accelerated the depletion of fossil-based resources. To address this challenge, it is essential to conduct in-depth research to develop biobased monomers as alternatives to petroleum-based ones [1,2,3]. Over recent decades, several aliphatic monomers, including ethylene glycol (EG), succinic acid (SA), 1,4-butanediol (BDO), 1,6-hexanediol (HDO), sebacic acid (SeA), lactic acid (LA), and 3-hydroxyalkanoic acids (3-HAs), have been successfully synthesized via biobased routes [4,5,6]. Accordingly, the development of these biobased monomers has driven the advancement of high-performance biobased polyesters, such as poly(butylene succinate) (PBS), poly(lactic acid) (PLA), poly(hydroxybutyrate) (PHB), and polyhydroxyalkanoates (PHAs) [7,8,9,10]. So far, these biobased alternatives are progressively substituting their petroleum-based products in different practical fields.

In addition to the above aliphatic monomers, some biobased aromatic monomers have also attracted significant research attention. Compared to aliphatic polyesters, aromatic polyesters generally exhibit superior mechanical strengths and higher glass transition temperatures (*T*_g_) [11]. Among numerous aromatic monomers, 2,5-furandicarboxylic acid (FDCA) and 2,5-thiophenedicarboxylic acid (TDCA), both containing five-membered heterocycles, have recently become the focus with regard to the design and development of sustainable polymers due to their unique molecular structures and properties [12,13,14,15].

Some FDCA-based polyesters have extensively been studied, such as poly(ethylene 2,5-furandicarboxylate) (PEF), poly(butylene-2,5-furan dicarboxylate) (PBF), and poly(hexene-2,5-furan dicarboxylate) (PHF) [16,17,18,19]. Notably, PEF demonstrates better mechanical properties than petroleum-derived poly(ethylene terephthalate) (PET), as well as superior thermal properties (a *T*_g_ value of 86 °C compared to 75 °C for PET) and barrier properties (the O_2_ transmission rate was reduced by 11 times, and the CO_2_ transmission rate was reduced by 19 times) [20,21,22]. These characteristics make PEF a promising PET alternative in packaging applications [23]. Similarly, some TDCA-based polymers, including poly(ethylene 2,5-thiophenedicarboxylate) (PETh), poly(butylene 2,5-thiophenedicarboxylate) (PBTh), and poly(propylene 2,5-thiophenedicarboxylate) (PPTh), have also recently been investigated [24,25,26]. Due to their structural similarity to that of furan-based polyesters, these materials also display excellent mechanical performances and notable gas barrier characteristics [27].

As a packaging material, PEF faces severe practical limitations due to its inherent brittleness and slow crystallization. Compared to PEF, PHF exhibits a higher elongation at break and faster crystallization [28,29]. However, to enhance its competitiveness in practical packaging applications, further modification of PHF is still necessary [30]. In a previous study, poly(hexamethylene-*co*-diethylene glycol furandicarboxylate) (PHDEGF) copolyesters were synthesized through a copolymerization process, with the aim of enhancing the mechanical and degradation properties of the PHF [31]. Mao et al. introduced 2,6-naphthalenedicarboxylic acid (NDCA) to PHF to synthesize a series of poly(hexamethylene 2,5-furandicarboxylate-*co*-2,6-naphthalate) copolyesters. While maintaining a certain mechanical strength, the *T*_g_ and barrier properties of the PHF were significantly improved [32]. Meng et al. synthesized amorphous poly(ethylene 2,5-thiophenedicarboxylate-*co*-2,5-furandicarboxylate) (PEThF) copolyesters using TDCA as a new comonomer. They demonstrated that the incorporation of TDCA concurrently enhanced PEF’s toughness while preserving its high *T*_g_, superior mechanical strength, and exceptional oxygen barrier performance [33]. In particular, the introduction of aromatic monomers effectively enhanced the mechanical properties of furan-based polyesters, thereby broadening their application scope.

To improve the toughness of the PHF without compromising its strength and gas barrier properties, we introduced biobased TDCA, as a new aromatic comonomer, to the main chain of the PHF in this research. Through a two-stage melt polymerization reaction, a series of biobased poly(hexamethylene furandicarboxylate-*co*-hexamethylene thiophenedicarboxylate) (PHFTh) copolyesters and their parent homopolyesters, PHF and poly(hexamethylene thiophenedicarboxylate) (PHTh), were synthesized. The chemical structures and compositions of the PHFTh copolyesters were first characterized. Subsequently, their thermal stabilities, thermal behaviors, crystal structures, and tensile mechanical properties were systematically investigated. The novelties of this study are as follows: On the one hand, fully biobased PHFTh copolyesters were synthesized for the first time to the best of our knowledge. On the other hand, compared to aliphatic comonomers, the introduction of the TDCA enabled a significant improvement in toughness while maintaining the inherent thermal stability and mechanical strength of the PHF. It is expected that the research results herein will be helpful for the development of novel biobased polyesters.

## 2. Experimental

### 2.1. Materials

Dimethyl furandicarboxylate (DMFD, 99%) was obtained from the Ningbo Institute of Materials Technology and Engineering, Chinese Academy of Sciences. HDO (99.5%) was purchased from Shanghai Macklin Biochemical Co., Ltd., Shanghai, China. TDCA (98%) was bought from Zhengzhou Alfa Chemical Co., Ltd., Zhengzhou, China. The catalyst tetrabutyl titanate (TBT, 99%) was acquired from Beijing Changping Jingxiang Chemical Factory, Beijing, China.

### 2.2. Synthesis of the PHFTh Copolyesters

**Scheme 1:**
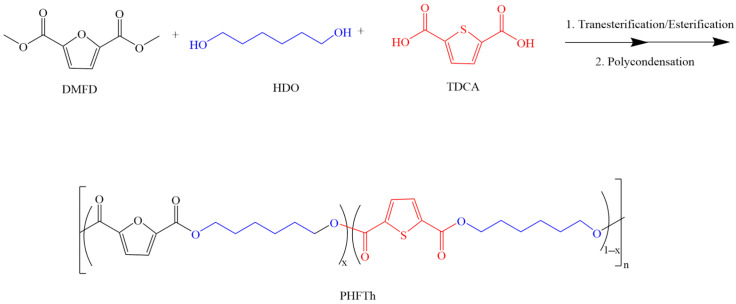
Synthesis route of PHFTh.

The PHFTh copolyesters were synthesized via a two-step melt polycondensation method, as shown in Scheme 1. DMFD, TDCA, and HDO were charged into a 250 mL three-necked flask at a molar ratio of ((DMFD + TDCA):HDO = 1:1.2), followed by the addition of 1 mL of TBT solution, at a concentration of 0.1 g/mL, as a catalyst. N_2_ was continuously introduced for 15 min while maintaining a mechanical stirring speed of 200–300 rpm. The mixture was first heated to 120 °C within 0.5 h. The transesterification/esterification was conducted by heating the mixture at a rate of 5 °C/min to 180 °C and holding it there for 2 h. Then, the temperature was increased at the same rate to 220 °C for an additional 2 h, until no further methanol/water was collected. For the polycondensation, the reactor was evacuated to below 200 Pa and heated to 230 °C for 3 h, until the polymer-climbing-rod phenomenon occurred. The resulting copolyesters were designated as PHFTh*x*, where *x* denoted the molar percentage of the TDCA in the feed. PHF and PHTh were synthesized in a similar procedure.

### 2.3. Characterization

The chemical structure and composition were determined on a Bruker AV600 hydrogen nuclear magnetic resonance (^1^H NMR) spectrometer (Bruker, Billerica, MA, USA) for PHF, PHTh, and PHFTh, using deuterated chloroform (CDCl_3_) as the solvent.

The intrinsic viscosity (*η*) values of all the synthesized polymers were measured in CDCl_3_ at a concentration of 0.4 g/dL on a Ubbelohde viscometer at 25 °C.

The thermal stabilities of all the samples were determined using thermogravimetric analysis (TGA) (TA Instrument Q50, New Castle, DE, USA). The experiments were carried out in a nitrogen atmosphere from room temperature to 580 °C at a heating rate of 20 °C/min.

The basic thermal parameters and nonisothermal crystallization behaviors were investigated on a differential scanning calorimeter (DSC) (TA Instrument DSC Q100, New Castle, DE, USA) at a nitrogen flow rate of 50 mL/min. Following 48 h of annealing at 25 °C, the samples were first cooled to −50 °C and then heated to 180 °C at 10 °C/min to investigate the melting behaviors. To measure the basis thermal parameter (*T*_g_), the samples were first heated to 180 °C at 40 °C/min and held for 3 min to erase the thermal history. Subsequently, they were rapidly cooled to −50 °C at 60 °C/min, followed by a heating to 180 °C at 10 °C/min. To study the melt crystallization behaviors, the samples were cooled to −50 °C at 10 °C/min and then heated to 180 °C at 10 °C/min.

Wide-angle X-ray diffraction (WAXD) experiments were performed using a Rigaku Ultima IV diffractometer (Rigaku, Tokyo, Japan) with CuKα radiation (λ = 1.5406 nm) at 40 kV and 200 mA. Scans were conducted from 5° to 50° at a scanning rate of 5°/min. All the samples were annealed for 12 h at specified temperatures: PHF at 115 °C; PHFTh10 and PHFTh30 at 100 °C; PHFTh50, PHFTh90, and PHTh at 80 °C; and PHFTh70 at 50 °C.

The tensile mechanical properties were measured on a universal tensile testing machine (UTM5205XHD from SUNS) at a crosshead rate of 20 mm/min at room temperature. The ISO 527-3:1995 standard was used in this research. The samples were prepared and cut using a hot-pressing process. The size of the dumbbell-shaped specimens was 1.0 mm in thickness, 4.0 mm in width, and 50.0 mm in length, at a standard distance of 20.0 mm. The average data were obtained from at least three tests.

## 3. Results and Discussion

### 3.1. Chemical Structure and Composition

**Figure 1:**
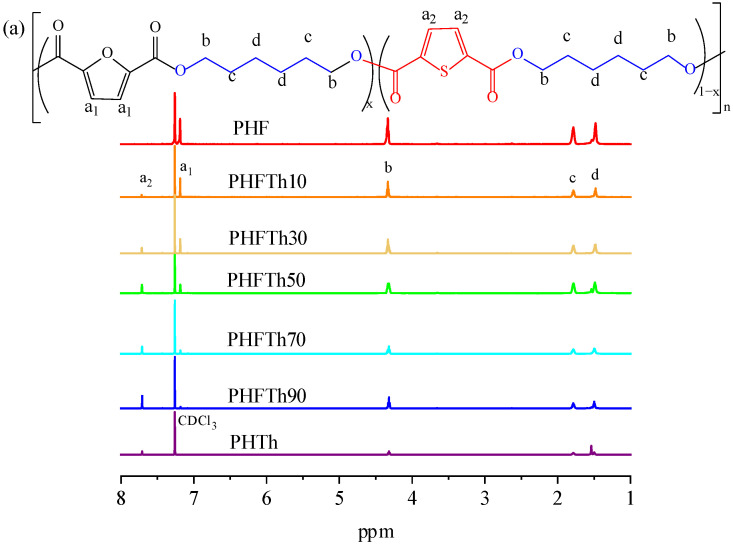

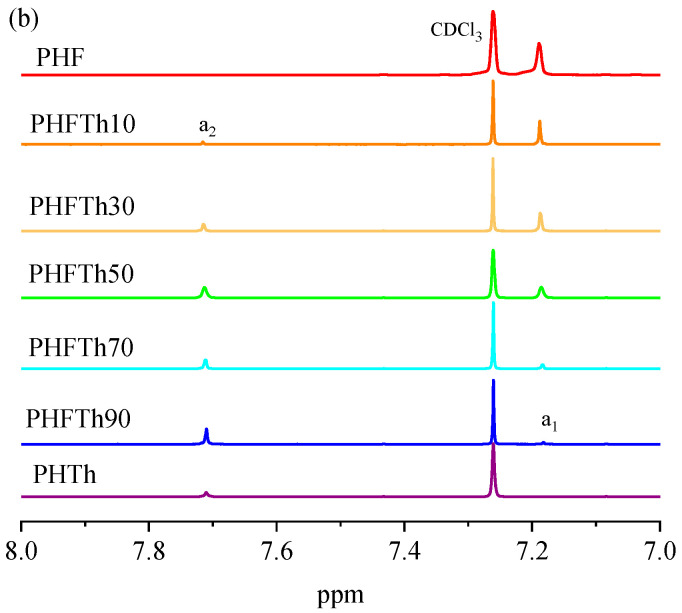
(**a**) Chemical structures and ^1^H NMR spectra of the PHF, PHTh, and PHFTh copolyesters and (**b**) enlargement of peaks *a*_1_ and *a*_2_.

Figure 1a shows the chemical structures and ^1^H NMR spectra of PHF, PHTh, and PHFTh. For PHF, the peak *a*_1_ at 7.19 ppm represented the protons in the furan. For PHTh, the peak *a*_2_ at 7.71 ppm corresponded to the protons in the thiophene structure. The peaks at 4.33 ppm (δH*^b^*), 1.79 ppm (δH*^c^*), and 1.48–1.50 ppm (δH*^d^*) represented the methylene protons (-CH_2_-) of HDO. Due to the similar chemical structures of furan and thiophene, the chemical shifts of the protons from HDO in the hexamethylene furandicarboxylate (HF) and HTh units were almost identical. As shown in Figure 1b, the actual composition ratio of the copolyesters was determined by calculating the integral area ratio of peak *a*_1_ to that of peak *a*_2_ using Equation (1).

(1) $$\Phi_{\mathrm{HTh}}=\frac{I_{a2}}{I_{a1}+I_{a2}}$$

where *I_a_*_1_ and *I_a_*_2_ represent the integral areas of chemical shifts *a*_1_ and *a*_2_, respectively. *Φ*_TDCA_ and *Φ*_HTh_ represent the feed ratio of the TDCA and the actual composition ratio of the HTh unit, respectively. Table 1 summarizes the results of the actual composition ratios of the PHFTh copolyesters. *Φ*_TDCA_ and *Φ*_HTh_ were close to each other, indicating that the composition of the PHFTh copolyesters could be simply controlled by adjusting the monomer feed ratio. The above results showed that PHFTh copolyesters were successfully synthesized.

The *η* values of the PHF, PHTh, and PHFTh copolyesters were calculated using Equation (2) and are presented in Table 1 [34].

(2) $$[\eta ]=\frac{\sqrt{2(\eta_{\mathrm{sp}}-\ln\eta_{r})}}{c}$$

where *η*_sp_ represents the specific viscosity, *η*_r_ represents the relative viscosity, and *c* is the concentration of the solution to be measured. The intrinsic viscosity (*η*) values of the PHF, PHTh, and PHFTh copolyesters measured in chloroform ranged from 0.63 to 0.76 dL/g.

### 3.2. Thermal Stability

**Figure 2:**
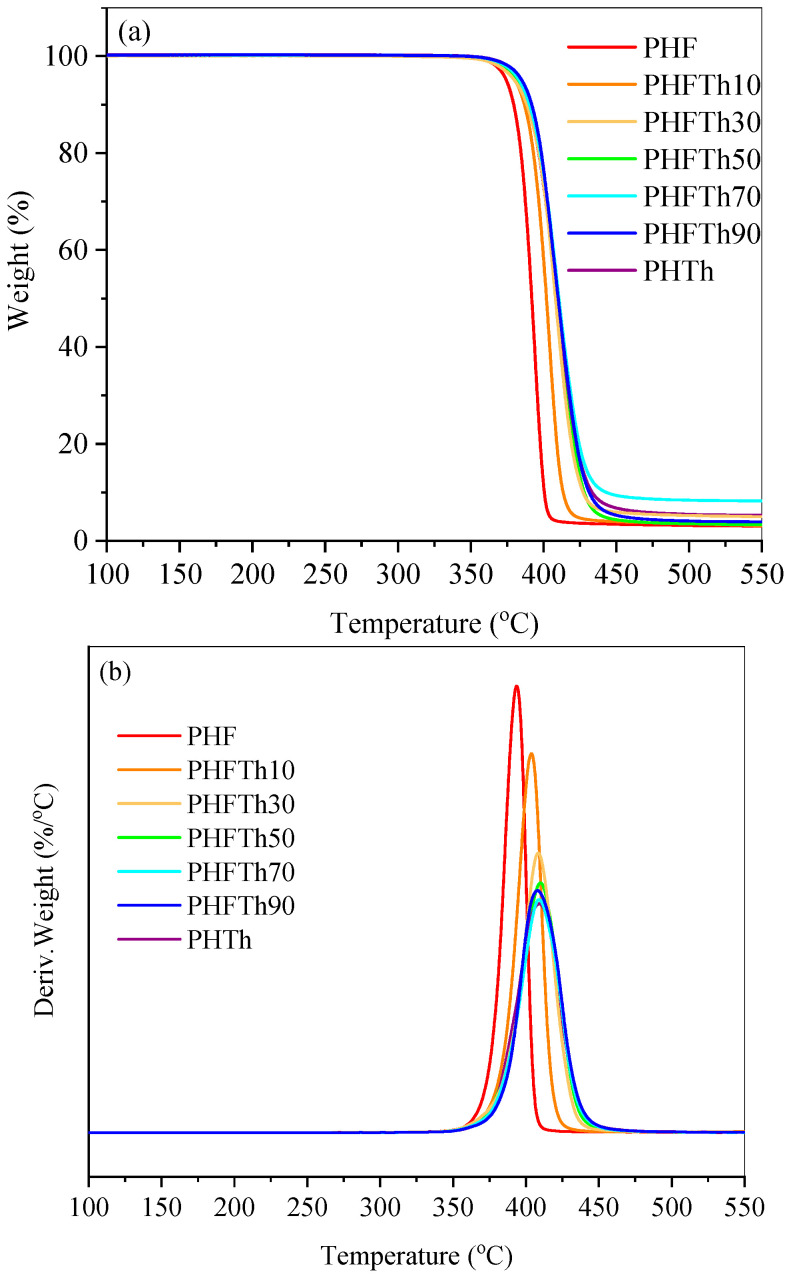
(**a**) TGA and (**b**) DTG curves of the PHF, PHTh, and PHFTh copolyesters.

The thermal stabilities of the PHF, PHTh, and PHFTh copolyesters were assessed with TGA. Figure 2 presents the TGA and corresponding differential TG (DTG) curves for the PHF, PHTh, and PHFTh copolyesters in a nitrogen atmosphere. As shown in Figure 2, both PHTh and PHFTh exhibited excellent thermal stabilities. The PHF, PHTh, and PHFTh copolyesters all underwent one-step degradation, with significant weight loss only occurring above 380 °C. The degradation temperature at a 5 wt.% weight loss (*T*_d_) and the temperature at the maximum decomposition rate (*T*_max_) values for PHF were 374.6 and 393.6 °C, respectively. For PHTh, the *T*_d_ and *T*_max_ values were 383.8 and 409.1 °C, respectively. The thermal stability of the PHTh was obviously higher than that of the PHF, which should be attributed to the difference in the chemical structures between TDCA and FDCA. From a chemical structure viewpoint, TDCA is more similar to terephthalic acid (TPA) than FDCA; therefore, TDCA-based polyesters displayed higher thermal stabilities than FDCA-based polyesters [35]. For instance, the *T*_d_ and *T*_max_ values of the PBF were 346 and 380 °C, respectively, which were lower than those of 352 and 386 °C for PBTh [35]. As a result, the *T*_d_ and *T*_max_ values of the PHFTh increased with increasing HTh unit content in this research. In sum, both the thiophene and furan structures may impart good thermal stability to PHFTh, which should be beneficial for melt processing.

### 3.3. Basic Thermal Parameters and Crystallization Behaviors

**Figure 3:**
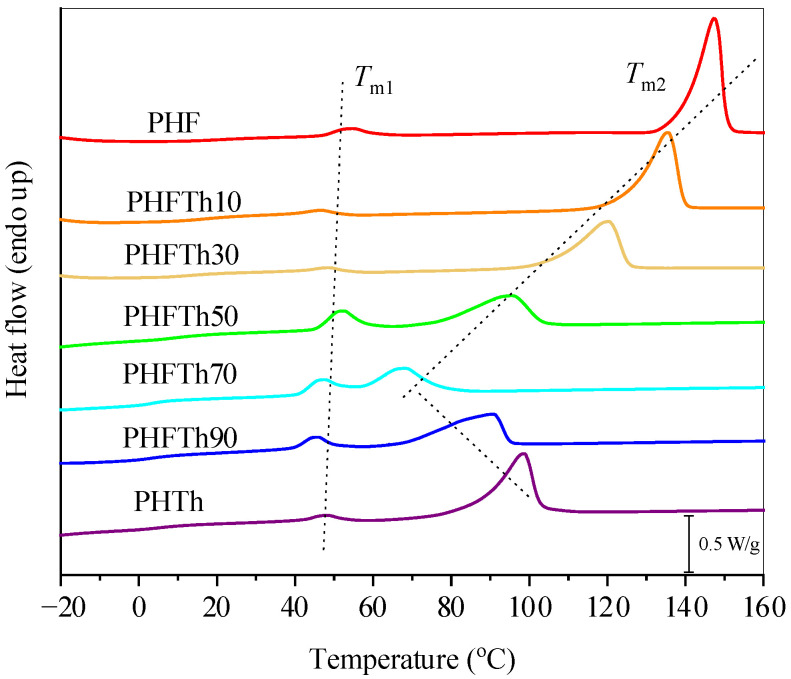
DSC curves of the PHF, PHTh, and PHFTh copolyesters heated at 10 °C/min after annealing at 25 °C for 48 h.

Figure 3 shows the DSC curves of all the synthesized polyesters heated after annealing at 25 °C for 48 h. As depicted in Figure 3, all the polyesters exhibited two melting-point endotherms. In the low-temperature region, the melting-point temperature at the low temperature (*T*_m1_) ranged from 46.7 to 54.5 °C, showing a melting enthalpy (Δ*H*_m_) of 1.7 to 7.0 J/g. This behavior was attributed to the development of a mesophase during the annealing process, which was associated with intermolecular hydrogen bonding [36,37]. A similar result was also reported by Lotti et al. in their recent research with regard to PHF-based multiblock copolyesters [38]. In the high-temperature region, the melting-point temperature at the high temperature (*T*_m2_) of the PHF was 147.5 °C, while that of the PHTh was 98.5 °C. With increasing HTh unit content, the *T*_m2_ value of the PHFTh copolyesters exhibited a trend of first decreasing and then increasing. PHFTh70 displayed the minimum *T*_m2_ value, corresponding to the so-called lowest eutectic melting point often found in isodimorphic random copolyesters [39,40]. However, the crystal structures of the copolyesters still need to be further studied to confirm this isodimorphic behavior in this research. The relevant data are summarized in Table 2. The processing window, defined as the temperature interval between the decomposition temperature and the melting point (*T*_d_—*T*_m_), significantly influenced the practical application of the polyesters. For instance, the processing window of the PHF was 227.1 °C, while that of the PHFTh30 was extended to 259.9 °C. These results demonstrated that the incorporation of the HTh unit substantially broadened the processing window of the copolyesters, thereby favoring their practical application from a polymer-processing viewpoint.

**Figure 4:**
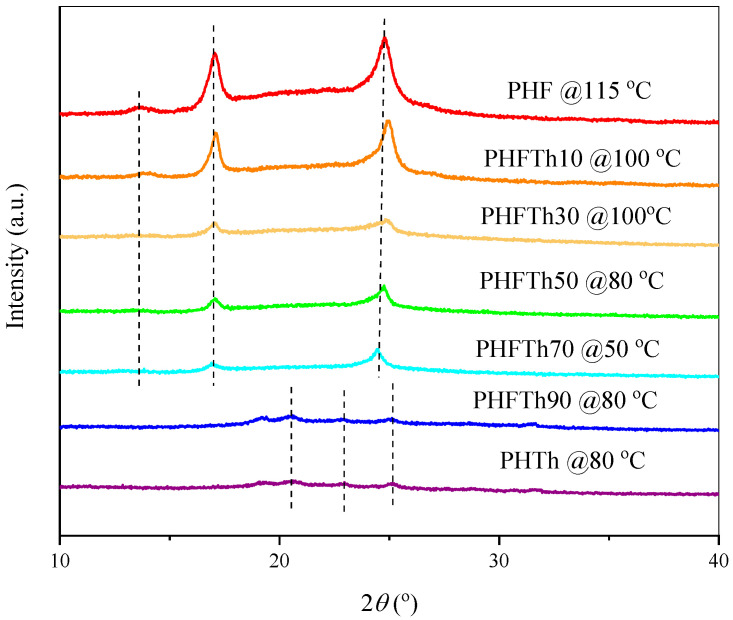
WAXD patterns of the PHF, PHTh, and PHFTh copolyesters at indicated temperatures.

In order to further resolve the attribution of the melting-point peaks that occurred after annealing, the crystal structures of the PHF, PHTh, and PHFTh copolyesters were further studied with WAXD. Figure 4 shows the WAXD patterns of the PHF, PHTh, and PHFTh copolyesters after isothermal crystallization for 10 h at specified temperatures. It could be concluded from Figure 4 that the PHF, PHTh, and PHFTh copolyesters were all semicrystalline polyesters, as they all showed diffraction peaks. PHF exhibited three strong characteristic diffraction peaks at 2θ = 13.7°, 17.0°, and 24.7°, while PHTh displayed three relatively weak diffraction peaks at 2θ = 20.7°, 22.9°, and 25.1°. The PHFTh10-70 copolyesters exhibited similar crystal structures to that of PHF, while the crystal structure of PHFTh90 was similar to that of the PHTh. The above results indicated that isodimorphism occurred in the PHFTh copolyesters in this research. In sum, depending on the HTh unit’s content, the PHFTh copolyesters showed the same crystal structure as that of PHF or that of PHTh. When the HTh unit content was 70 mol% and below, the PHFTh copolyesters crystallized through the same crystal structure as that of PHF. On the contrary, the copolyester displayed the same crystal structure as that of PHTh when the HTh unit’s content was 90 mol% and above. From the above DSC and WAXD results, it could also be concluded that the crystallization ability of the PHF was stronger than that of the PHTh, as evidenced by both the higher *T*_m_ and the stronger diffraction peaks.

**Figure 5:**
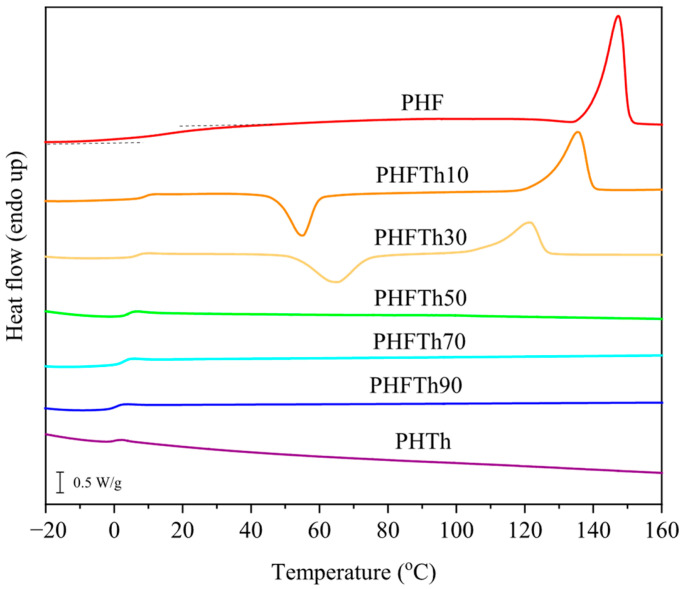
DSC curves of the PHF, PHTh, and PHFTh copolyesters heated at 10 °C/min after quenching at 60 °C/min.

One of the most basic thermal parameters, *T*_g_, of all the samples was further investigated with DSC. From Figure 5, the *T*_g_ of the PHF was 15.6 °C, while that of the PHTh was −0.1 °C. In the literature, the *T*_g_ of the PBF was 39.8 °C, while that of the PBTh was 25.3 °C [35]; moreover, the *T*_g_ of the PPF was 52 °C, while that of the PPTh was 40 °C [41]. Therefore, as the content of the HTh unit increased, the *T*_g_ of the copolyesters gradually decreased. As also illustrated in Figure 5, only PHFTh10 and PHFTh30 exhibited cold crystallization peaks at 54.9 and 65.1 °C, respectively. Due to the fast melt crystallization of the PHF, it could still crystallize rapidly at a cooling rate of 60 °C/min. On the contrary, the crystallization rates of the PHFTh50-90 and PHTh were too slow to crystallize at a heating rate of 10 °C/min, thus their melting peaks were not observed.

**Figure 6:**
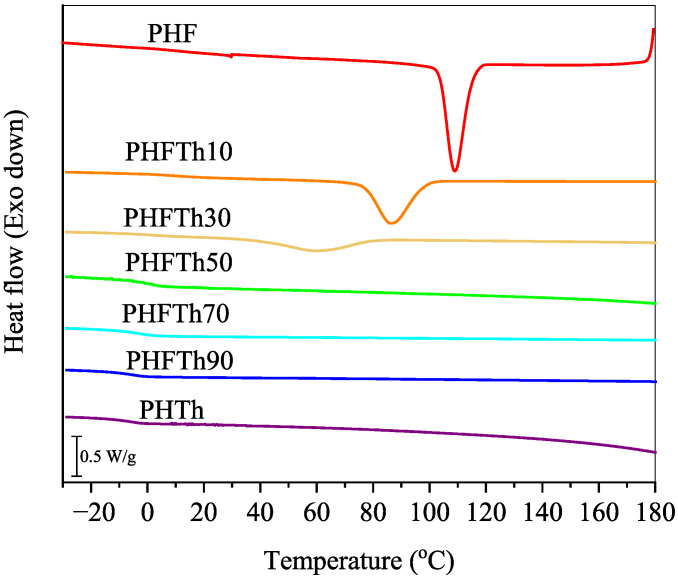
DSC curves of the PHF, PHTh, and PHFTh copolyesters cooled at 10 °C/min.

The melt crystallization behaviors were further studied. Figure 6 presents the DSC curves of the PHF, PHTh, and PHFTh copolyesters cooled at 10 °C/min. The melt crystallization temperature (*T*_cc_) and crystallization enthalpy (Δ*H*_cc_) for PHF were 109.0 °C and 59.6 J/g, respectively. However, no crystallization peak was observed for PHTh due to its weak crystallization ability. For PHFTh10 and PHFTh30, as the HTh unit’s content gradually increased, both the *T*_cc_ and Δ*H*_cc_ values decreased; moreover, the crystallization peak gradually became broader. However, when the HTh unit’s content exceeded 30 mol%, it significantly inhibited the crystallization of the HF phase. Therefore, PHFTh50 and PHFTh70 were not able to crystallize, even at this cooling rate.

**Figure 7:**
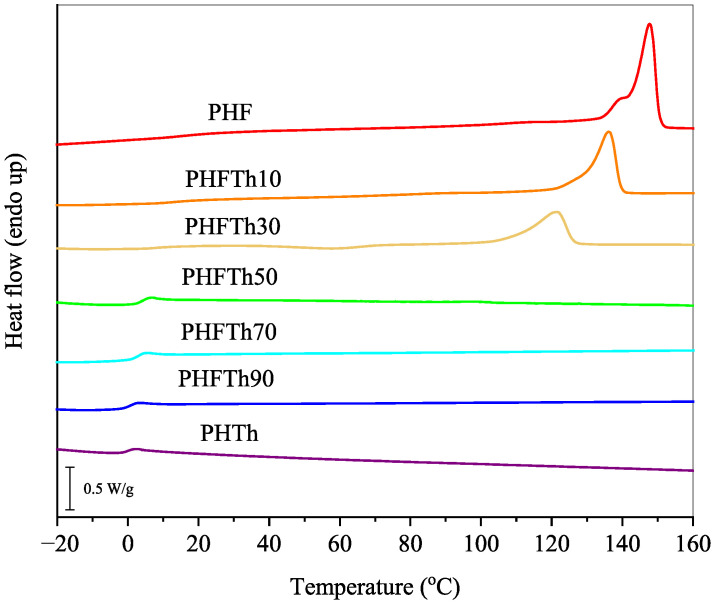
DSC curves of the PHF, PHTh, and PHFTh copolyesters heated at 10 °C/min after a cooling process at 10 °C/min.

Figure 7 displays the subsequent DSC curves of the PHF, PHTh, and PHFTh copolyesters heated at 10 °C/min after the cooling process. The cold crystallization peak of the PHFTh10 disappeared, confirming complete crystallization during the melt crystallization process. However, PHFTh30 still showed a cold crystallization peak at 58.1 °C, indicating incomplete melt crystallization. The *T*_m_ and Δ*H*_m_ values of the PHF were 147.7 °C and 66.0 J/g, respectively. For PHFTh10 and PHFTh30, both *T*_m_ and Δ*H*_m_ progressively decreased with increasing HTh unit content. Even the cooling rate was only 10 °C/min, no cold crystallization or melting peaks were observed for PHFTh50-90 and PHTh, which is consistent with the results in Figure 5. The relevant thermal parameters are summarized in Table 3 for comparison.

### 3.4. Mechanical Properties

**Figure 8:**
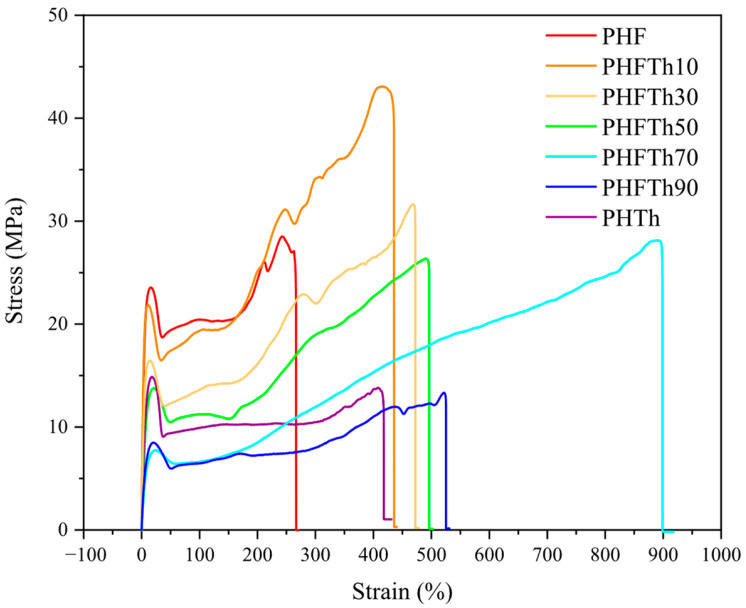
Stress–strain curves of the PHF, PHTh, and PHFTh copolyesters.

Figure 8 displays the stress–strain curves of the PHF, PHTh, and PHFTh copolyesters. The fluctuations shown in Figure 8 were attributed to the stress oscillation behavior, which is very common in polyesters [42]. PHF demonstrated a relatively lower elongation at break (*ε*_b_) but a higher Young’s modulus (*E*_t_) and a higher tensile strength (*σ*_b_) than those of PHTh. This mechanical behavior originated from the weaker dipole moment of the C-S-C bond compared to that of the C-O-C bond, which weakened the intermolecular interactions and enhanced the flexibility of the chain. Compared to PHF, PHFTh10 showed significant increases in *σ*_b_ and *ε*_b_ with almost no decrease in *E*_t_. With the increasing number of HTh segments, the *σ*_b_ and *E*_t_ values of the PHFTh copolyesters showed decreasing trends. Among all the PHFTh copolyesters, PHFTh70 reached the minimum values of *E*_t_ (115.5 ± 3.8 MPa). However, the *ε*_b_ value of the PHFTh70 reached as high as 875.8 ± 31.1%, which was more than three times that of the PHF. The detailed mechanical property data are provided in Table 4.

## 4. Conclusions

In this study, a series of novel biobased PHFTh copolyesters and their parent homopolyesters, PHF and PHTh, were successfully synthesized via two-stage melt polycondensation. ^1^H NMR confirmed that the actual composition ratio of the copolyesters was close to the feed ratio of the monomers. The synthesized PHFTh copolyesters exhibited excellent thermal stabilities, displaying a broad processing window. The DSC and WAXD results revealed that the PHFTh copolyesters were semicrystalline in nature for all the compositions, as long as suitable crystallization conditions were used (i.e., the annealing time or crystallization time was sufficiently long for the samples to crystallize). In addition, the PHFTh copolyesters displayed a typical isodimorphism phenomenon, with PHFTh70 exhibiting the lowest eutectic melting point. When the HTh unit content was 70 mol% and below, the copolyesters displayed the same crystal structures as that of the PHF. On the contrary, when the HTh unit content was 90 mol% and above, the crystal structure of the copolyester was the same as that of the PHTh. However, the melt crystallization and subsequent melting behavior studies at 10 °C/min indicated that only PHF, PHFTh10, and PHFTh30 were able to crystallize, as evidenced by the melt crystallization exothermic peak and subsequent melting endothermic peak. Under the same conditions, the rest of the copolyesters and PHTh were not able to crystallize due to their weak crystallization abilities. Additionally, with increases in the HTh unit’s content, the *T*_g_ of the copolyesters gradually decreased, indicating that the chain mobility was enhanced. Therefore, the introduction of the HTh significantly improved the *ε*_b_ value of the PHFTh. For instance, PHFTh70 achieved a remarkable *ε*_b_ value of 875.8 ± 31.1% while still maintaining a relatively high *σ*_b_ value of 27.7 ± 1.4 MPa. In sum, novel fully biobased PHFTh copolyesters possessing tunable thermal behaviors and mechanical properties were synthesized in this research, which should be interesting and important in the development of the biobased polyester field.

## Figures and Tables

**Table 1:** The compositions and intrinsic viscosity (*η*) values of the PHF, PHTh, and PHFTh copolyesters.

Sample	*Φ*_TDCA_ (mol%)	*Φ*_HTh_ (mol%)	*η* (dL/g)
PHF	0	0	0.63
PHFTh10	10	10.0	0.74
PHFTh30	30	28.2	0.76
PHFTh50	50	49.8	0.69
PHFTh70	70	69.9	0.74
PHFTh90	90	89.3	0.70
PHTh	100	100	0.72

**Table 2:** DSC data of the PHF, PHTh, and PHFTh copolyesters after annealing at 25 °C for 48 h.

Sample	*T*_m1_ (°C)	Δ*H*_m1_ (J/g)	*T*_m2_ (°C)	Δ*H*_m2_ (J/g)
PHF	54.5	3.4	147.5	47.2
PHFTh10	46.7	2.1	135.4	39.8
PHFTh30	48.6	1.7	120.3	30.5
PHFTh50	51.7	7.0	95.5	25.7
PHFTh70	46.6	4.3	68.2	12.7
PHFTh90	45.3	3.7	90.8	25.9
PHTh	48.1	2.3	98.5	36.3

**Table 3:** Thermal properties of the PHF, PHTh, and PHFTh copolyesters.

Sample	*T*_g_ (°C)	*T*_cc_ (°C)	Δ*H*_cc_ (J/g)	*T*_m_ (°C)	Δ*H*_m_ (J/g)	*T*_d_ (°C)	*T*_max_ (°C)
PHF	12.3	109.0	59.6	147.7	66.0	374.6	393.6
PHFTh10	9.7	86.5	31.1	136.1	32.7	380.5	403.8
PHFTh30	6.4	59.4	19.5	121.5	25.1	380.2	408.4
PHFTh50	4.2	-	-	-	-	382.1	410.0
PHFTh70	3.3	-	-	-	-	383.2	409.0
PHFTh90	1.1	-	-	-	-	385.0	408.1
PHTh	−0.1	-	-	-	-	383.8	409.1

**Table 4:** Mechanical properties of the PHF, PHTh, and PHFTh copolyesters.

Sample	*E*_t_ (MPa)	*σ*_b_ (MPa)	*ε*_b_ (%)
PHF	565.6 ± 23.1	30.3 ± 1.4	261.6 ± 8.3
PHFTh10	558.5 ± 22.0	40.4 ± 1.4	448.7 ± 21.0
PHFTh30	443.4 ± 16.4	31.8 ± 0.6	488.4 ± 23.4
PHFTh50	208.5 ± 24.6	24.7 ± 1.4	481.1 ± 14.6
PHFTh70	115.5 ± 3.8	27.7 ± 1.4	875.8 ± 31.1
PHFTh90	167.3 ± 16.6	12.4 ± 0.9	498.9 ± 23.7
PHTh	248.1 ± 10.0	12.8 ± 1.0	435.9 ± 18.7

## Data Availability

The original contributions presented in this study are included in the article. Further inquiries can be directed to the corresponding author.
